# Phagocytic receptors activate and immune inhibitory receptor SIRPα inhibits phagocytosis through paxillin and cofilin

**DOI:** 10.3389/fncel.2014.00104

**Published:** 2014-04-16

**Authors:** Miri Gitik, Rachel Kleinhaus, Smadar Hadas, Fanny Reichert, Shlomo Rotshenker

**Affiliations:** ^1^Department of Medical Neurobiology, Institute for Medical Research Israel–Canada, Faculty of Medicine, Hebrew UniversityJerusalem, Israel; ^2^Brain Disease Research Center, Institute for Medical Research Israel–Canada, Faculty of Medicine, Hebrew UniversityJerusalem, Israel

**Keywords:** microglia, macrophage, phagocytosis, CD47, SIRPα, paxillin, cofilin, cytoskeleton

## Abstract

The innate immune function of phagocytosis of apoptotic cells, tissue debris, pathogens, and cancer cells is essential for homeostasis, tissue repair, fighting infection, and combating malignancy. Phagocytosis is carried out in the central nervous system (CNS) by resident microglia and in both CNS and peripheral nervous system by recruited macrophages. While phagocytosis proceeds, bystander healthy cells protect themselves by sending a “do not eat me” message to phagocytes as CD47 on their surface ligates immune inhibitory receptor SIRPα on the surface of phagocytes and SIRPα then produces the signaling which inhibits phagocytosis. This helpful mechanism becomes harmful when tissue debris and unhealthy cells inhibit their own phagocytosis by employing the same mechanism. However, the inhibitory signaling that SIRPα produces has not been fully revealed. We focus here on how SIRPα inhibits the phagocytosis of the tissue debris “degenerated myelin” which hinders repair in axonal injury and neurodegenerative diseases. We tested whether SIRPα inhibits phagocytosis by regulating cytoskeleton function through paxillin and cofilin since (a) the cytoskeleton generates the mechanical forces that drive phagocytosis and (b) both paxillin and cofilin control cytoskeleton function. Paxillin and cofilin were transiently activated in microglia as phagocytosis was activated. In contrast, paxillin and cofilin were continuously activated and phagocytosis augmented in microglia in which SIRPα expression was knocked-down by SIRPα-shRNA. Further, levels of phagocytosis, paxillin activation, and cofilin activation positively correlated with one another. Taken together, these observations suggest a novel mechanism whereby paxillin and cofilin are targeted to control phagocytosis by both the activating signaling that phagocytic receptors produce by promoting the activation of paxillin and cofilin and the inhibiting signaling that immune inhibitory SIRPα produces by promoting the inactivation of paxillin and cofilin.

## INTRODUCTION

Phagocytosis, the engulfment and internalization of large particles by phagocytes, is an innate immune function which is carried out in the central nervous system (CNS) parenchyma by resident microglia and in both CNS parenchyma and peripheral nervous system (PNS) nerves by recruited bone marrow-derived macrophages. Phagocytosis is essential for homeostasis as it clears apoptotic and aging cells, tissue repair when it removes tissue debris, fighting infection since it scavenges pathogens and combating malignancy while it eliminates cancer cells. During such phagocytosis, bystander healthy cells are protected from being phagocytosed. CD47-SIRPα interactions provide this protection when CD47 on the surface of healthy cells binds and activates the immune inhibitory receptor signal regulatory protein-α (SIRPα; also known as CD172α and SHPS-1) on the surface of phagocytes and SIRPα then produces the signaling that inhibits phagocytosis (Barclay and Brown, 2006; Matozaki et al., 2009; Oldenborg, 2013). Such observations led to define CD47 as a marker of “self” which sends a “do not eat me” message to phagocytes (Oldenborg et al., 2000).

This protective mechanism becomes detrimental under pathological conditions when tissue debris (Gitik et al., 2011) and malignant cells (Chao et al., 2012; Kim et al., 2012) employ CD47-SIRPα interactions to inhibit their own clearance. We (Gitik et al., 2011) and others thereafter (Han et al., 2012) documented in this regard that the tissue debris “degenerated myelin” inhibits its own phagocytosis as CD47 on degenerated myelin ligates SIRPα on macrophages and microglia. Intact myelin is a specialized extension of Schwann cells in the PNS and oligodendrocytes in the CNS, and further, it surrounds the larger diameter of PNS and CNS axons, enabling them fast conduction of electrical activity. Myelin breaks down in Wallerian degeneration following traumatic axonal injury (Waller, 1850; Vargas and Barres, 2007; Rotshenker, 2011) and in neurodegenerative diseases such as multiple sclerosis (Stadelmann and Bruck, 2008). The rapid phagocytosis of the tissue debris degenerated myelin so produced is essential since it impedes repair and exacerbates disease by arresting the regeneration of severed adult axons (David and Aguayo, 1981), preventing remyelination (Kotter et al., 2006) and advancing the production of membrane attack complexes that damage nearby intact tissue (Mead et al., 2002). Thus, understanding how SIRPα inhibits the phagocytosis of degenerated myelin is of utmost importance.

Previous studies documented that SIRPα signaling involves the recruitment of tyrosine phosphatases SHP-1 and SHP-2 to the cytoplasmic domain of SIRPα, and subsequently, SHP-1 and SHP-2 may dephosphorylate phosphotyrosine sites in their immediate downstream target molecules (Barclay and Brown, 2006; Matozaki et al., 2009; Oldenborg, 2013). However, the molecular events further downstream to the SIRPα/SHP-1/2 complex have not been fully elucidated.

The phagocytosis of degenerated myelin is mediated by phagocytic receptors complement receptor-3 (CR3), scavenger receptor SRA-I/II (SRA) and Fcγ receptor (FcγR) (Reichert et al., 2001; Reichert and Rotshenker, 2003; Rotshenker, 2003). CR3 ligates both unopsonized and C3bi-opsonized degenerated myelin (i.e., opsonized by complement protein C3bi), SRA ligates unopsonized degenerated myelin, and FcγR ligates IgG-opsonized degenerated myelin (i.e., opsonized by anti-myelin antibodies). Further, degenerated myelin can simultaneously ligate several receptors. We focus in this study on the cytoskeleton as a potential target through which SIRPα may inhibit the phagocytosis of degenerated myelin which is mediated mostly by CR3, to a lesser level by SRA, and not at all by FcγR since phagocytosis was assayed in the absence of anti-myelin antibodies.

We previously documented that CR3 and SRA mediated phagocytosis of degenerated myelin involves structural changes in phagocytes. First, filopodia and lamellipodia extend and engulf the myelin-debris, and then, filopodia/lamellipodia retract, and pull-in the myelin-debris into the phagocyte where it is degraded (Hadas et al., 2012). Extension and retraction of membrane protrusions are driven by mechanical forces that are generated by the cytoskeleton. The production of membrane protrusions depends on the remodeling of filamentous actin (F-actin); i.e., the breakdown of old filaments and the production of new ones (Wang et al., 2007; Bernstein and Bamburg, 2010). Cofilin/ADF (actin depolymerizing factor) is a family of proteins that controls F-actin remodeling. Active unphosphorylated cofilin advances the remodeling F-actin and thereby the production of filopodia/lamellipodia whereas inactive phosphorylated cofilin (p-cofilin) stabilizes F-actin and thereby reduces the production of filopodia/lamellipodia. We previously documented in this regard that (a) filopodia/lamellipodia are involved in the phagocytosis of degenerated myelin (Hadas et al., 2012); (b) active unphosphorylated cofilin advances whereas inactive p-cofilin reduces phagocytosis (Hadas et al., 2012); (c) the small GTPase RhoA, which signals through ROCK, stabilizes F-actin, and further down-regulates phagocytosis (Gitik et al., 2010). The retraction of filopodia/lamellipodia can be driven by mechanical forces that are generated by contraction which is based on the interaction between F-actin and non-muscle-myosin in which motor activity was triggered (Clark et al., 2007; Vicente-Manzanares et al., 2009). We previously documented in this regard that the phagocytosis of degenerated myelin is advanced by myosin light chain kinase (MLCK) after it triggers motor activity in non-muscle-myosin which then leads to F-actin/non-muscle-myosin-based contraction (Gitik et al., 2010).

**FIGURE 1:**
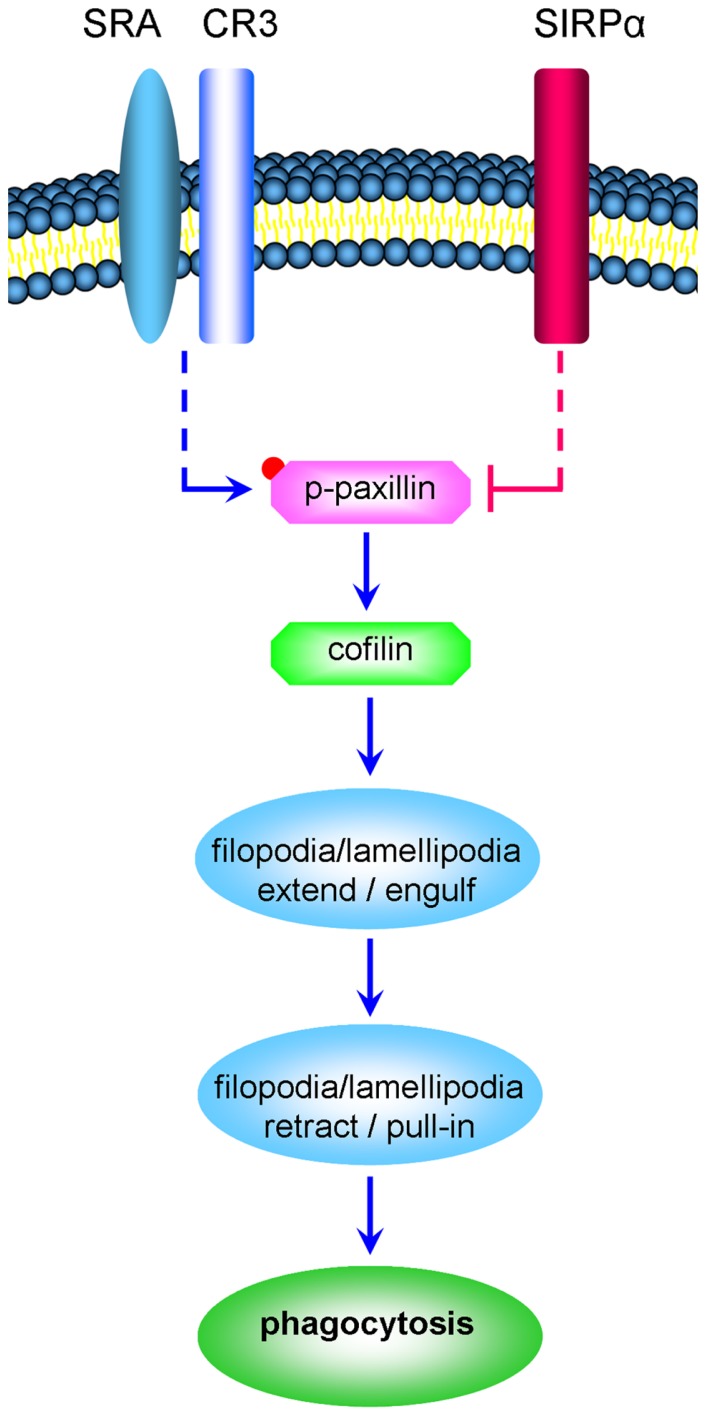
**CR3 and SRA advance the phagocytosis of degenerated myelin by promoting the activation of paxillin and cofilin whereas SIRPα inhibits phagocytosis by promoting the inactivation of paxillin and cofilin (a schematic representation of the working hypothesis).** Degenerated myelin ligates simultaneously phagocytic receptors CR3 and SRA and immune inhibitory receptor SIRPα. CR3 and SRA produce the signaling that culminates in structural changes (marked by ellipses); filopodia and lamellipodia first engulf the myelin-debris as they extend and then pull-in the myelin-debris as they retract. We hypothesize the following. Phagocytic receptors promote paxillin activation through phosphorylation (paxillin → p-paxillin) leading to cofilin activation through dephosphorylation (p-cofilin → cofilin). In turn, cofilin promotes the production of filopodia and lamellipodia and so phagocytosis is advanced. In contrast, SIRPα promotes the inactivation of paxillin through p-paxillin dephosphorylation (p-paxillin → paxillin) leading to cofilin inactivation through phosphorylation (cofilin → p-cofilin). In turn, the production of filopodia and lamellipodia is reduced and so phagocytosis is inhibited. Retraction of filopodia and lamellipodia is promoted by MLCK triggering motor activity in non-muscle-myosin which then initiates F-actin/non-muscle-myosin-based contraction (not illustrated). Dashed lines mark indirect interactions; blue lines mark activation and red lines inhibition of phagocytosis.

Taken that (a) active cofilin advances the phagocytosis of degenerated myelin by CR3 (an αM/β2 integrin; Hadas et al., 2012), (b) integrins promote the activation the scaffold/adaptor protein paxillin through focal-adhesion-kinase (FAK) and Src and, (c) paxillin in its active phosphorylated state (p-paxillin) can indirectly activate cofilin (Deakin and Turner, 2008), we hypothesize that SIRPα could inhibit phagocytosis by promoting the inactivation of both paxillin and cofilin (**Figure 1**). Observations made in this study support this working hypothesis, further suggesting that phagocytosis is determined by the balance between CR3 and SRA advancing phagocytosis by promoting paxillin and cofilin activation and SIRPα inhibiting phagocytosis by promoting paxillin and cofilin inactivation.

## MATERIALS AND METHODS

### ANIMALS

Animals Balb/C mice that were obtained from Harlan (Israel) were handled in accordance with the national research council guide for the care and use of laboratory animals and the approval of the institutional committee.

### PRIMARY MICROGLIA

Primary microglia were isolated from brains of neonate mice as previously described (Reichert and Rotshenker, 2003). In brief, brains were stripped of their meninges, enzymatically dissociated, cells plated on poly-L-lysine coated flasks for 1 week, replated for 1–2 h on bacteriological plates and non-adherent cells washed away. The vast majority (over 95%) of adherent cells are microglia judged by morphology and positive immunoreactivity to Galectin-3/MAC-2, CR3, and F4/80 (Reichert and Rotshenker, 1996, 1999)

### GENERATION OF MICROGLIA WITH STABLE REDUCED SIRPα EXPRESSION

Generation of microglia with stable reduced SIRPα expression was previously described and documented by us (Gitik et al., 2011). Reduction of SIRPα expression was achieved through lentiviral infection of wild-type Balb/C microglia with short hairpin RNAs directed against mouse SIRPα mRNA (SIRPα-shRNA) using pLKO.1 puro plasmids (Sigma, Israel). Three different shRNA sequences were used. All were effective in reducing SIRPα expression (60, 70, and 90% of levels in wild-type microglia). The one used in this study, as in our previous study (Gitik et al., 2011), was the SIRPα cDNA coding sequence 5′CCGGTGGTTC-AAAGAACTCGAGTTCTTGCCCATCTTTGAACCATTTTTG-3′. The plasmid was transfected into a 293T-based packaging cell line, and the resulting culture supernatant was used for lentiviral infection. Infected microglia were selected on the basis of their resistance to puromycin brought by the pLKO.1 plasmid. Levels of SIRPα protein expression were monitored by immuno blot analysis. As a control, microglia were infected in a similar way with the shRNA sequence 5′CTTACGCTGAGTACTTCGA-3′ against the non-target firefly Luciferase gene. We refer to these microglia as control microglia in text and Control Luciferase (Con-Luc) microglia in figures.

### MYELIN ISOLATION

Myelin isolation from mouse brains has been previously described (Slobodov et al., 2001) and also visualized (Gitik et al., 2011). The isolated myelin is “degenerated myelin” since isolation involves breakdown of intact myelin.

### PHAGOCYTOSIS OF DEGENERATED MYELIN

Phagocytosis of degenerated myelin was assayed in microglia that were plated in 96-well tissue culture plates at a density that minimizes cell-cell contact (0.25-1.5 × 10^4^/well) in the presence of Dulbecco’s Modified Eagle Medium (DMEM)/F12 supplemented by 10% FCS. Non-adherent microglia were washed out after 2 h and adherent microglia left to rest overnight. Next, phagocytes were washed and degenerated myelin added in the presence of serum for the indicated time periods. Then, unphagocytosed degenerated myelin was washed out and levels of phagocytosis determined by enzyme linked immunosobent assay (ELISA).

### ELISA ASSAY TO QUANTIFY THE PHAGOCYTOSIS OF DEGENERATED MYELIN

ELISA assay to quantify the phagocytosis of degenerated myelin is based on the detection of myelin basic protein (MBP) in microglia lysates. Since MBP is unique to myelin and is not produced by phagocytes, MBP levels detected in phagocyte cytoplasm are proportional to levels of degenerated myelin that is phagocytosed. In brief, after non-phagocytosed degenerated myelin is washed away and remaining degenerated myelin has been phagocytosed/internalized, phagocytes are lysed (0.05 M carbonate buffer, pH 10), lysates transferred to high protein absorbance plates (Nalge Nunc International, NY, USA) and levels of MBP determined by ELISA using anti-MBP monoclonal antibody. A detailed protocol is given in (Slobodov et al., 2001) where we also determined that more than 95% of the detected MBP arises from phagocytosed/internalized degenerated myelin. We further verified the validity of this phagocytosis assay by documenting over 95% inhibition of the phagocytosis of degenerated myelin in the presence of cytochalasin-D (not shown).

### QUANTIFICATION OF PHAGOCYTOSIS

Quantification of phagocytosis was carried out in the following way. When phagocytosis by SIRPα-KD microglia (i.e., microglia in which SIRPα expression was knocked-down with SIRPα-shRNA) was compared to phagocytosis by control microglia (i.e., microglia infected with non-target Luciferase-shRNA), phagocytosis by each population was first normalized to the number of respective microglia counted in 1 mm^2^ areas at the center of wells. Normalizing phagocytosis to cell number is required since SIRPα-KD and control microglia may differ in their adherence properties, thus resulting in different number of adherent microglia even when the same number of cells was initially seeded. To this end, microglia in replicate plates were fixed (i.e., instead of being lysed for the phagocytosis assay), stained, and counted. Phagocytosis normalized to cell number by control microglia was defined 100% and phagocytosis normalized to cell number by SIRPα-KD microglia was calculated as percentage of phagocytosis by control microglia. Statistical analysis was carried out as detailed in figure legends.

### IMMUNOBLOT ANALYSIS

Immunoblot analysis Microglia were plated in 10 cm tissue culture plates at a density that minimizes cell–cell contact (3 × 10^6^ cells per plate) in the presence of DMEM supplemented by 10% FCS, and left to rest overnight. Phagocytes were washed in fresh DMEM supplemented by 10% FCS, degenerated myelin added in the presence of serum for the indicated time periods and unphagocytosed degenerated myelin washed out. For lysis, microglia were washed in PBS and lysed in ice cold lysis buffer (Tris HCL 1 M pH 7.5, MgCl_2_ 1 M, NaCl 4 M, 0.5% NP-40, 0.1% DTT, and 0.1% NaVa) supplemented with protease and phosphatase inhibitors cocktail (Sigma–Aldrich, Israel), cellular debris was removed by centrifugation, and total protein content determined using Bradford reagent (Sigma–Aldrich, Israel). Equal protein content from whole cell lysates was separated on SDS-PAGE to detect SIRPα, paxillin, cofilin, and GAPDH. Proteins were blotted to nitrocellulose membranes, blocked with 10% non-fat milk or 5% BSA in TBS (Tris-buffered saline) for 1 h at RT, incubated over night at 4°C in the presence of primary antibodies mouse anti-rat SIRPα/CD172α (Serotec, Oxford, England), mouse anti-human GAPDH, rabbit anti-cofilin, rabbit anti-pS^3^-cofilin-1, and rabbit anti-pY^118^-paxillin (Santa Cruz Biotechnology, USA), rabbit anti-paxillin (Cell Signaling, USA), and mouse anti-actin monoclonal antibody (MP biomedicals, CA, USA). Blots were washed with TBST and incubated with respective secondary antibodies goat anti-rat, goat anti-rabbit, and goat anti-mouse conjugated to HRP (Jackson ImmunoReserach, USA) for 40-min at RT. Proteins were visualized with EZ-ECL kit for HRP detection (Beit Haemek, Israel). The intensities of immunoblot bands were determined by ImageJ software and quantification and statistical analysis was carried out as detailed in figure legends.

## RESULTS

### SIRPα PROMOTES THE INACTIVATION OF COFILIN

We previously documented the following with regard to the phagocytosis of degenerated myelin by microglia in the absence of anti-myelin antibodies: (a) phagocytosis is mostly mediated by CR3 and to a lesser degree by SRA (Rotshenker, 2003), (b) phagocytosis is advanced by cofilin (Hadas et al., 2012), (c) phagocytosis is inhibited by SIRPα (Gitik et al., 2011). Thus, SIRPα could inhibit phagocytosis by promoting the inactivation of cofilin. In this case, reducing SIRPα in phagocytes is expected to promote the activation of cofilin by shifting the balance from inactive p-cofilin (cofilin phosphorylated at serine site S^3^) to active unphosphorylated cofilin (cofilin) concurrent with augmenting phagocytosis. We addressed this issue by examining p-cofilin levels before and during phagocytosis in microglia in which SIRPα expression was knocked-down (SIRPα-KD) by lentiviral infection with SIRPα-shRNA, and further, in control microglia that were similarly infected with the non-target firefly Luciferase-shRNA.

**FIGURE 2:**
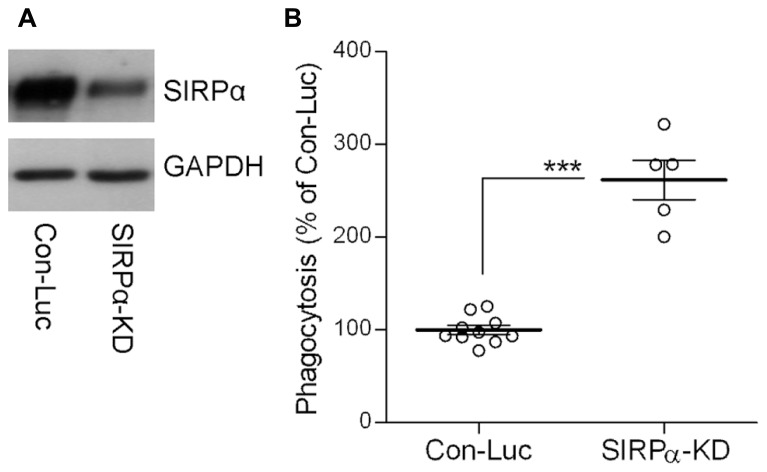
**The phagocytosis of degenerated myelin is augmented in SIRPα-KD microglia. (A)** A representative (one of three) immunoblot of SIRPα and GAPDH. SIRPα levels are reduced in Balb/C microglia infected with SIRPα-shRNA (SIRPα-KD) compared with control microglia infected with non-target Luciferase-shRNA (Con-Luc). **(B)** Phagocytosis of degenerated myelin is augmented in SIRPα-KD microglia compared with phagocytosis by control microglia (Con-Luc). Phagocytosis by SIRPα-KD microglia was calculated as a percentage of phagocytosis by control microglia that was defined as 100%. Values of individual experiments each performed in triplicates and averages ±SE are given. Significance of difference by Mann–Whitney is ***p < 0.001.

**FIGURE 3:**
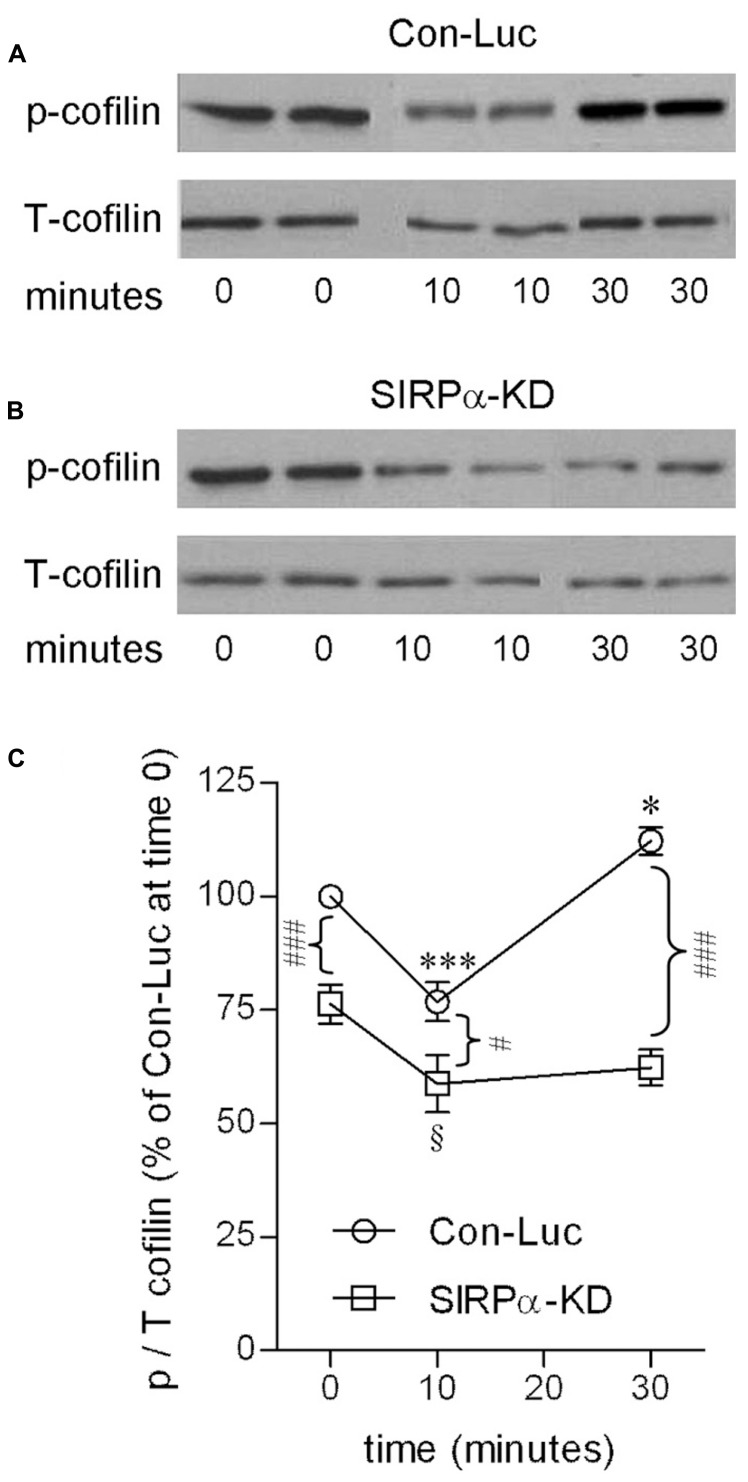
**Cofilin activation is transient in phagocytosing control microglia but continuous in phagocytosing SIRPα-KD microglia.** Immunoblot analysis of phosphorylated and total cofilin-1 (p- and T-cofilin) in **(A)** control (Con-Luc) and **(B)** SIRPα-KD microglia before the onset of phagocytosis (time 0), and after 10 and 30 min of phagocytosis. The antibodies used for immunoblot analysis identify cofilin and cofilin-1 that is phosphorylated at S^3^. **(C)** Quantitation of the ratio p/T based on immunoblot analysis. The ratio p/T in non-phagocytosing Con-Luc microglia (i.e., at time 0) was defined 100%. Then, p/T in all other non-phagocytosing and phagocytosing microglia was calculated as percentage of p/T in non-phagocytosing Con-Luc microglia. Average values ±SEM of four to six experiments, each performed in duplicates, are given. Significance of differences between initial values at 0 min and those at 10 and 30 min by one way ANOVA and the Dunnet post test are *p < 0.05 and ***p < 0.001 for Con-Luc microglia and ^§^*p* < 0.05 for SIRPα-KD microglia. Significance of difference between Con-Luc and SIRPα-KD microglia by two way ANOVA and the Bonferroni post test are ^#^*p* < 0.05 and ^###^*p* < 0.001. Significance of difference between 10 and 30 min in Con-Luc microglia by one way ANOVA and the Tukey’s post test is *p* < 0.001 (not marked).

Indeed, SIRPα levels were reduced (**Figure 2A**) and phagocytosis was augmented (**Figure 2B**) in SIRPα-KD microglia compared with control microglia, thus confirming our previously reported observations (Gitik et al., 2011). Levels of p-cofilin were then determined by immunoblot analysis using an antibody raised against cofilin that is phosphorylated at serine site S^3^ (**Figures 3A,C**). Levels of p-cofilin were reduced in control microglia after 10 min of phagocytosis down to about 75% of those in non-phagocytosing control microglia. Then, after 30 min of phagocytosis, levels of p-cofilin returned to and rose above their initial levels in non-phagocytosing control microglia. These findings confirm our previously reported observations (Hadas et al., 2012).

SIRPα-KD microglia differed from control microglia with respect to p-cofilin levels before and during phagocytosis (**Figures 3B,C**). Levels of p-cofilin were reduced in non-phagocytosing SIRPα-KD microglia down to about 75% of those in non-phagocytosing control microglia. After 10 and 30 min of phagocytosis, p-cofilin levels were reduced further down to about 60% of those in non-phagocytosing control microglia. Taken together, cofilin was transiently activated during prolonged phagocytosis in control microglia but continuously activated in SIRPα-KD microglia, suggesting that normally SIRPα promotes the inactivation of cofilin through serine (S^3^) phosphorylation.

### SIRPα PROMOTES THE INACTIVATION OF PAXILLIN

Taken that the SIRPα/SHP-1/2 complex dephosphorylates phosphotyrosine sites in its immediate target molecules (Barclay and Brown, 2006; Matozaki et al., 2009; Oldenborg, 2013) and our present findings that SIRPα promotes cofilin inactivation by serine phosphorylation, SIRPα could not have inactivated cofilin directly. However, SIRPα could inactivate cofilin indirectly through paxillin. This proposition is based on previous observations that paxillin is activated by tyrosine phosphorylation (paxillin phosphorylated at tyrosine site Y^118^), and further, that p-paxillin can indirectly activate cofilin (Deakin and Turner, 2008). Thus if SIRPα promotes the inactivation of paxillin, then levels of active p-paxillin are expected to be higher in SIRPα-KD microglia than in control microglia.

**FIGURE 4:**
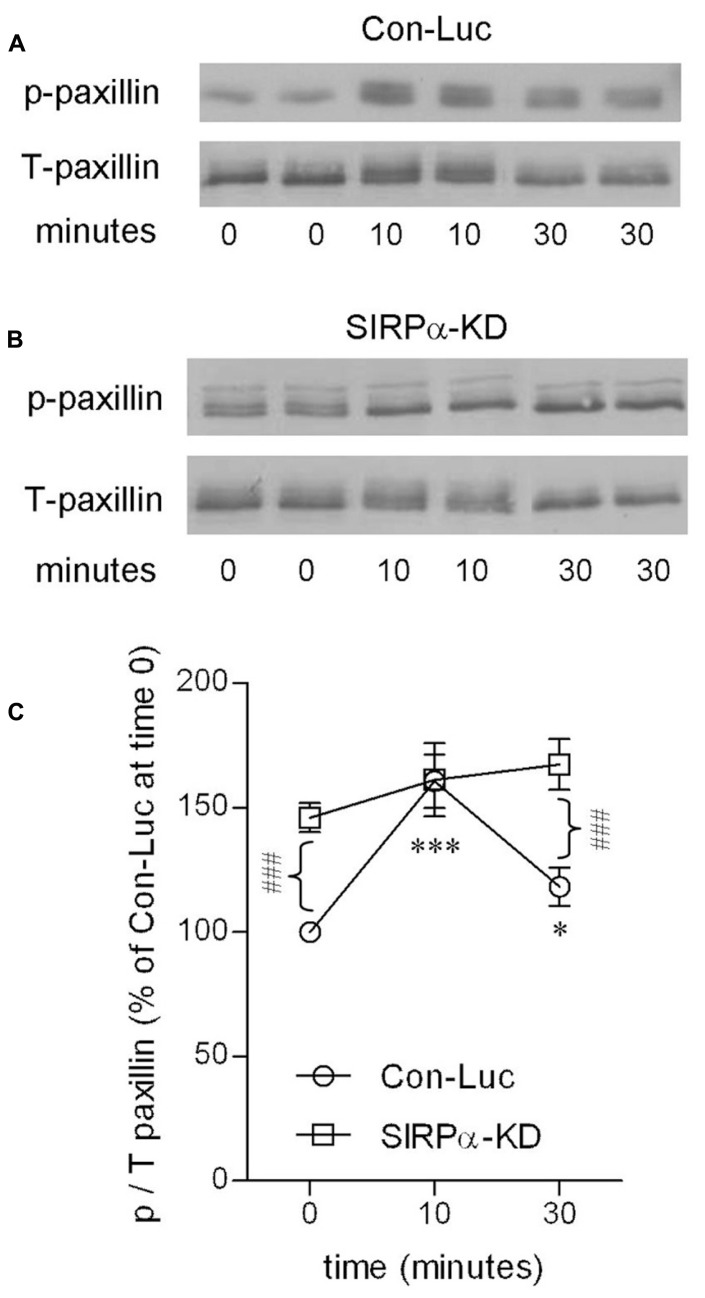
**Paxillin activation is transient in phagocytosing control microglia but continuous in phagocytosing SIRPα-KD microglia.** Immunoblot analysis of p- and T-paxillin in **(A)** control (Con-Luc), and **(B)** SIRPα-KD microglia before the onset of phagocytosis (time 0), and after 10 and 30 min of phagocytosis. The antibodies used for immunoblot analysis identify paxillin and paxillin that is phosphorylated at Y^118^. **(C)** Quantitation of the ratio p/T based on immunoblot analysis. The ratio p/T in non-phagocytosing Con-Luc microglia (i.e., at time 0) was defined 100%. Then p/T in all other non-phagocytosing and phagocytosing microglia was calculated as percentage of p/T in non-phagocytosing Con-Luc microglia. Average values SEM of four to eight experiments, each performed in duplicates, are given. Significance of differences between initial values at 0 min and those at 10 and 30 min by one way ANOVA and the Dunnet post test are *p < 0.05 and ***p < 0.001 for Con-Luc microglia. Significance of difference between Con-Luc and SIRPα-KD microglia by two way ANOVA and the Bonferroni post test is ^###^*p* < 0.001. Significance of difference between 10 and 30 min in Con-Luc microglia by one way ANOVA and the Tukey’s post test is *p* < 0.01 (not marked).

Levels of p-paxillin were determined by immunoblot analysis using an antibody raised against paxillin which is phosphorylated at tyrosine site Y^118^ (**Figures 4A,C**). Levels of p-paxillin increased in control microglia to about 160% of those in non-phagocytosing control microglia after 10 min of phagocytosis. Then, after 30 min of phagocytosis, p-paxillin levels decreased significantly to about 120% of those in non-phagocytosing control microglia.

SIRPα-KD microglia differed from control microglia with respect to p-paxillin levels before and during phagocytosis (**Figures 4B,C**). Levels of p-paxillin were about 145% higher in non-phagocytosing SIRPα-KD microglia than in non-phagocytosing control microglia. After 10 and 30 min of phagocytosis, levels of p-paxillin increased further to about 160 and 170% of those in non-phagocytosing control microglia. Taken together, paxillin was transiently activated during prolonged phagocytosis in control microglia and continuously activated in SIRPα-KD microglia, suggesting that normally SIRPα promotes the inactivation of paxillin through phosphotyrosine (pY^118^) dephosphorylation.

### PAXILLIN ACTIVATION, COFILIN ACTIVATION AND PHAGOCYTOSIS POSITIVELY CORRELATE WITH ONE ANOTHER

**FIGURE 5:**
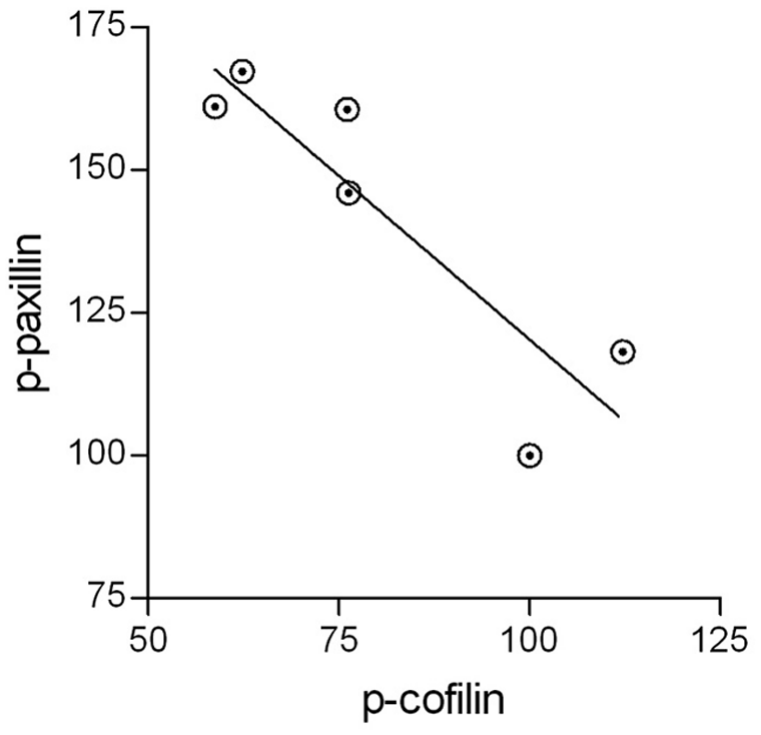
**The activation of paxillin and cofilin are linked.** All values of active p-paxillin and inactive p-cofilin that are presented in **Figures 3** and **4** were subjected to linear regression and correlation analysis. The two display a negative correlation with an *r*^2^ value of 0.79. Significance of correlation by the Pearson double tailed test is *p* < 0.05.

It has been suggested that active p-paxillin can indirectly activate cofilin by promoting the transition from inactive p-cofilin to active unphosphorylated cofilin (Deakin and Turner, 2008). If this is the case during the phagocytosis of degenerated myelin, then levels of active p-paxillin and levels of inactive p-cofilin should negatively correlate with one another during phagocytosis, and further, phagocytosis augmentation should positively correlate with the activation of both paxillin and cofilin. Indeed, control microglia displayed transient increases in p-paxillin and transient decreases in p-cofilin during phagocytosis, and further, SIRPα-KD microglia exhibited higher levels of p-paxillin and lower levels of p-cofilin compared with control microglia both before and throughout phagocytosis which was augmented in SIRPα-KD microglia compared with control microglia. To obtain a quantitative measure to these apparent correlations, all values of p-paxillin and p-cofilin presented in **Figures 3** and **4** were subjected to linear regression and correlation analysis (**Figure 5**). Levels of p-paxillin and p-cofilin displayed significant (*p* < 0.05) negative correlation with an *r*^2^ value of 0.79. Thus paxillin activation (reflected by higher levels of p-paxillin) and cofilin activation (reflected by lower levels of p-cofilin) positively correlated with one another. Since levels of phagocytosis increased in SIRPα-KD microglia compared with control microglia at the same time as levels of both paxillin and cofilin activation increased, all three (i.e., levels of phagocytosis, paxillin activation, and cofilin activation) positively correlated with one another.

## DISCUSSION

Observations made in this study suggest a novel mechanism whereby paxillin and cofilin are targeted to control the phagocytosis of degenerated myelin by both the activating signaling which phagocytic receptors CR3 and SRA produce and the inhibiting signaling that immune inhibitory receptor SIRPα produces. In this regard, phagocytic receptors advance phagocytosis by promoting the activation of paxillin and cofilin whereas immune inhibitory receptor SIRPα inhibits phagocytosis by promoting the inactivation of the two.

The activation of phagocytosis by paxillin and cofilin is suggested by the findings that phagocytosis positively correlates with the activation of both paxillin and cofilin in both control and SIRPα-KD microglia. In control microglia, paxillin and cofilin were activated concurrent with the activation of phagocytosis, and in SIRPα-KD microglia, levels of paxillin and cofilin activation increased simultaneously with the increase in phagocytosis.

The role cofilin plays in the phagocytosis of degenerated myelin was recently reported and discussed by us (Hadas et al., 2012). Cofilin advances phagocytosis by promoting the remodeling of F-actin and thereby the production of filopodia/lamellipodia which engulf the myelin-debris. We presently suggest that cofilin activation is promoted by paxillin. This proposition is based on the observations that paxillin can indirectly activate cofilin (Deakin and Turner, 2008) and our present findings that levels of paxillin activation and levels of cofilin activation positively correlated with one another. The involvement of p-paxillin in signaling phagocytosis by CR3 and SRA which is documented here could also take place in signaling phagocytosis by FcγR since paxillin is tyrosine phosphorylated during FcγR mediated phagocytosis (Greenberg et al., 1994).

That SIRPα inhibits the phagocytosis of degenerated myelin by promoting the inactivation of both paxillin and cofilin is suggested by the synchronized occurrence of three events in SIRPα-KD microglia. First, phagocytosis is augmented in SIRPα-KD microglia compared with control microglia; second, the activation of both paxillin and cofilin increases in non-phagocytosing as well as in phagocytosing SIRPα-KD microglia compared with non-phagocytosing and phagocytosing control microglia; third, the kinetics of the activation of both paxillin and cofilin switched from transient in phagocytosing control microglia to continuous in phagocytosing SIRPα-KD microglia. SIRPα dependent inactivation of paxillin and cofilin was not reported before for any of the functions that SIRPα is involved in. We further raise the possibility that SIRPα could directly inactivate paxillin through dephosphorylation since paxillin is activated through tyrosine phosphorylation (Deakin and Turner, 2008) and the SIRPα/SHP-1/2 complex dephosphorylates phosphotyrosine sites (Barclay and Brown, 2006; Matozaki et al., 2009; Oldenborg, 2013).

In conclusion, the phagocytosis of degenerated myelin is determined by the balance between the signaling produced by CR3 and SRA which activates paxillin and cofilin and the signaling produced by SIRPα which inactivates paxillin and cofilin. SIRPα could also target paxillin and cofilin while inhibiting phagocytosis which is mediated by additional phagocytic receptors such as FcγR (Oldenborg et al., 2001) and during the phagocytosis of particles other than myelin-debris such as aging red blood cells (Oldenborg et al., 2000) and tumor cells (Chao et al., 2012; Kim et al., 2012).

## Conflict of Interest Statement

The authors declare that the research was conducted in the absence of any commercial or financial relationships that could be construed as a potential conflict of interest.
